# Selective oxidative modification of tryptophan and cysteine residues using visible light responsive Rh doped SrTiO_3_ photocatalyst

**DOI:** 10.1038/s41598-025-04870-z

**Published:** 2025-07-01

**Authors:** Sho Usuki, Naoko Taki, Yuma Uesaka, Haru Togawa, Shanhu Liu, Kenji Yamatoya, Kazuya Nakata

**Affiliations:** 1 Graduate School of Bio-Applications and Systems Engineering, Tokyo University of Agriculture and Technology, 2-24-16 Naka-cho, Koganei, Tokyo, 184-0012 Japan; 2 Henan Joint International Research Laboratory of Environmental Pollution Control Materials, Henan Key Laboratory of Polyoxometalate Chemistry, College of Chemistry and Chemical Engineering, Henan University, Kaifeng, 475004 People’s Republic of China; 3 Laboratory of Genomic Function Engineering, Department of Life Sciences, School of Agriculture, Meiji University, 1-1-1 Higashimita, Tama-Ward, Kawasaki, Kanagawa 214-8571 Japan

**Keywords:** Side chain modification, Visible light-responsive photocatalyst, g-STO:Rh, Peptide engineering

## Abstract

In recent years, amino acids and peptides have attracted significant attention in food and medical fields due to their functionality. These functionalities largely depend on the chemical properties of their side chains, and efficient methods for selective side chain modification are desired. In this study, we investigated the selective modification of amino acids and peptides using rhodium-doped SrTiO_3_ (g-STO:Rh), a visible light-responsive photocatalyst. HPLC and LCMS analyses revealed that g-STO:Rh exhibited selective reactivity toward tryptophan and cysteine among the 20 protein-constituent amino acids. While cysteine was oxidatively dimerized to cystine, tryptophan underwent selective oxidation of its indole ring side chain, forming *N*-formylkynurenine. Furthermore, studies on dipeptides and tripeptides containing tryptophan have demonstrated that selective oxidation proceeds similarly to tryptophan residues within peptides.

**Supplementary Information:**

The online version contains supplementary material available at 10.1038/s41598-025-04870-z.

## Introduction

Amino acids and peptides have garnered widespread attention across various fields, not only for their importance as fundamental building units in biological systems but also for their diverse potential applications^1–3^. Amino acids are industrially utilized as ligands for transition metal catalysts and as components of polymer materials^4–6^, while also playing crucial roles as substrates in peptide synthesis^7,8^ and in the design of physiologically active molecules for drug development^9,10^. Peptides, on the other hand, are being developed as food additives^11,12^ and pharmaceuticals due to their specific physiological activities^13,14^. The chemical properties of side chains play a decisive role in the functional expression of these molecules^15^. For instance, research has revealed that age-related functional decline of proteins is attributed to oxidative modifications of side chains^16,17^, which has emerged as a significant focus in aging research. Furthermore, chemical modification of side chains is recognized as an effective means to either impart new functions to amino acids and peptides or to control their existing functions^18–20^. As side chain modifications play an essential role in controlling the functions of amino acids and peptides, there is a strong demand for the development of efficient and selective modification methods.

Photocatalysts are materials that convert light energy into chemical reactions energy and are applied across a wide range of fields, including environmental purification and chemical synthesis^21,22^. Their reaction mechanism begins when light with energy greater than the bandgap energy is irradiated. This light irradiation excites electrons from the valence band to the conduction band, creating holes in the valence band. The generated electrons and holes possess reducing and oxidizing abilities, respectively, and induce redox reactions in the chemicals on or near the photocatalyst surface. In these photocatalytic reactions, reduction reactions by excited electrons and oxidation reactions by holes proceed simultaneously. Particularly, in the presence of water and oxygen, these electrons and holes generate reactive oxygen species, which are known to cause powerful redox reactions. This characteristic enables various chemical transformations, such as the decomposition of organic compounds and oxidation of surface functional groups^23–26^. Furthermore, photocatalytic methods offer advantages, as they can proceed under ambient temperature and pressure conditions and do not require complex reagents.

Modification of amino acids using photocatalysts has attracted attention as an environmentally friendly chemical method^27,28^. Various amino acid transformations have been reported particularly through photocatalytic reactions using TiO_2_. For instance, oxidation reactions of alanine, asparagine, aspartic acid, serine, and histidine have been confirmed^29^. Additionally, the conversion of aspartic acid and serine using silver-loaded TiO_2_ has been reported^30^. Elsellami et al. investigated the photocatalytic oxidation of tryptophan by TiO_2_, resulting in the formation of *N*-formylkynurenine and kynurenine^31^. Szabó-Bárdos et al. reported the photocatalytic conversion of phenylalanine in aqueous solution in the presence of TiO_2_, which produces L-2-hydroxyphenylalanine and L-dihydroxyphenylalanine^32^. However, in TiO_2_ photocatalytic reactions, non-specific oxidative modifications occur regardless of amino acid type due to the generation of large amounts of reactive oxygen species along with holes. This non-specificity is a general characteristic of not only TiO_2_ but also other photocatalysts, which is advantageous when proceeding with multiple reactions simultaneously. On the other hand, the development of methods for selective chemical modification of specific amino acids has not progressed sufficiently. Against this background, the visible light-responsive photocatalyst ground Rh-doped SrTiO_3_ (g-STO:Rh) has been reported to show interesting characteristics. In the presence of *Escherichia coli* and bacteriophage *Qβ*, it exhibits selective reactivity different from that observed with TiO_2_^33^, and this selectivity has been suggested to involve electrostatic interactions^34^. Since amino acid side chains are thought to be deeply involved in these electrostatic interactions, g-STO:Rh may possess selective modification capability for specific amino acid side chains.

Previous studies on amino acid modification using photocatalysts have primarily focused on non-specific oxidation reactions using conventional photocatalysts such as TiO_2_. Although g-STO:Rh has been suggested to exhibit selective reactivity through electrostatic interactions, its reactivity towards amino acids has not yet been thoroughly investigated. In this study, we conducted a comprehensive investigation of the photocatalytic effects of g-STO:Rh on the 20 amino acids present in biological systems. In particular, we examined the possibility of selective modification of the side chains of each amino acid in detail. Furthermore, for amino acids that showed selective modification, we verified whether similar modifications occur in dipeptides and tripeptides containing these amino acids, and explored the potential for application to longer peptide chains. In this study, we aimed to establish a selective amino acid modification method using g-STO:Rh.

## Experimental

### Synthesis of g-STO:Rh

g-STO:Rh powder was synthesized according to the following procedure. SrCO_3_ (Kanto Chemical Co., Inc.) 2.53 g, TiO_2_ (Soekawa Chemical Co., Ltd.) 1.27 g, and Rh_2_O_3_ (FUJIFILM Wako Pure Chemical Corporation) 20.3 mg were mixed with a pestle in an alumina mortar for 4–5 h. The obtained mixed powder was heated in an electric furnace (Denken-Highdental Co., Ltd.) to 900 °C at a rate of 9.7 °C min^-1^ and calcined for 1 h. After cooling, it was further heated to 1,100 °C at a rate of 7.2 °C min^-1^ and calcined for 10 h. After cooling, the powder was ground at 800 rpm for 60 min using a planetary ball mill (FRITSCH), filtered by suction using a 0.2 µm membrane filter (Merck Millipore), and dried at 60 °C for 24 h.

### Treatment of amino acids with g-STO:Rh

g-STO:Rh (45 mg) was dispersed in 15 mL of ultrapure water by sonication for 10 min. Each amino acid was added to this suspension to achieve a final concentration of 1 mM to prepare the reaction solution. The reaction was conducted by irradiation with a 200 W Xenon lamp (300 nm ~ 800 nm) through a UV cut filter (Y-44, λ > 440 nm). The light intensity was adjusted to 110 mW cm^-2^. Control experiments were performed with light irradiation alone without g-STO:Rh, and with g-STO:Rh in the dark. After specific time intervals, the reaction solution was collected and centrifuged at 9,000 × g for 5 min to collect the supernatant. A 500 µL sample was collected at 0 h before starting the reaction, and samples were subsequently collected every 1 h after starting light irradiation. The amino acid concentration in the reaction solution was measured using LCMS. The analysis was performed using an Intrada Amino Acid column (50 × 3 mm) under the following conditions: Two mobile phases were used for the analysis. Mobile phase A consisted of acetonitrile and formic acid (100:0.1), whereas mobile phase B was 100 mM ammonium formate. The following gradient elution program was employed: mobile phase B was maintained at 14% for 0–5 min, increased linearly from 14 to 100% during 5–12 min, decreased linearly from 100 to 14% during 12–14 min, and maintained at 14% for 14–16 min. Mobile phase A was used at percentages complementary to mobile phase B throughout the gradient program. The flow rate was 0.6 mL min^-1^ and the column temperature was maintained at 35 °C. Ionization was performed in ESI positive mode, and measurements were conducted using Q3 scan and Q3SIM. For the quantitative analysis of *N*-formylkynurenine, commercial standard material was not readily available. Therefore, isolated *N*-formylkynurenine was employed as the reference standard to establish the calibration curve for analytical quantification. Sulfuric acid was measured using an ion chromatography conductivity detector (CDD-10A VP, Shimadzu). Shimadzu Shim-pack IC-SA2 (stationary phase: quaternary ammonium group, size: 4.0 × 250 mm) was used for the analysis, and the temperature was maintained at 40 ºC. The mobile phase consisted of a mixed solution of 12 mM NaHCO_3_ and 0.6 mM Na_2_CO_3_, measured under the conditions of a flow rate of 1.0 mL min^−1^.

The conversion rate was calculated using the following equation: Conversion rate (%) = 100 (%) − (Amino acid concentration after each irradiation time/Amino acid concentration at 0 h irradiation) × 100 (%).

### ^1^H and ^13^C-NMR measurements

The following experiments were conducted to isolate the products for ^1^H and ^13^C-NMR measurements. After the photocatalytic reaction with 10 mL of 10 mM l-tryptophan solution, the sample was concentrated to 1 mL using a rotary evaporator and then separated by preparative HPLC. The separation was performed using a Intrada Amino Acid column (50 × 3 mm) with 14% acetonitrile/86% 100 mM ammonium formate (v/v) as the mobile phase at a flow rate of 0.6 mL min^-1^ at 35 °C. The solvent in the collected samples was evaporated using a rotary evaporator, followed by drying under nitrogen flow. The dried samples were dissolved in deuterated solvent for NMR analysis. ^1^H NMR spectrum was recorded at 500 MHz using a JNM-ECA500 (JEOL). ^13^C NMR spectrum was recorded at 125 MHz using a JNM-ECA500 (JEOL).

### Treatment of peptides with g-STO:Rh

A suspension was prepared by adding 45 mg of g-STO:Rh to 15 mL of ultrapure water and dispersing it through sonication for 10 min. Each peptide was added to this suspension at a final concentration of 1 mM to create the reaction solution. The reaction was conducted by irradiating with a 200 W Xenon lamp through a UV cut filter (Y-44, λ > 440 nm). The light intensity was adjusted to 110 mW cm^-2^. Control experiments were performed with light irradiation alone without g-STO:Rh, and with g-STO:Rh under dark conditions. Subsequently, peptide solutions were collected at specified time intervals and centrifuged at 9,000 × g for 5 min to obtain the supernatant. A 500 µL sample was collected at 0 h before initiating the reaction, followed by sample collection every 1 h after commencing light irradiation. Analysis of peptides in the reaction solution was performed using LCMS. The analysis was conducted using an Intrada Amino Acid column (50 × 3 mm) under the following conditions: Two mobile phases were used for the analysis. Mobile phase A consisted of acetonitrile and formic acid (100:0.3), whereas mobile phase B consisted of acetonitrile and 100 mM ammonium formate (60:40). The following gradient elution program was employed: mobile phase B was increased linearly from 70 to 100% during 0–6 min, maintained at 100% during 6–15 min, and decreased linearly from 100 to 70% during 15–17 min. Mobile phase A was used at percentages complementary to mobile phase B throughout the gradient program. The flow rate was maintained at 0.6 mL min^-1^, and the column temperature was set at 37 °C. The injection volume was 5 µL. Ionization was performed in ESI positive mode, and measurements were conducted using Q3 scan and Q3SIM.

### Zeta potential measurements

To evaluate the surface charge of g-STO:Rh particles, zeta potential measurements were conducted using a ZETASIZER Nano ZS (Malvern Panalytical). g-STO:Rh was added at 30 mg (3 mg mL^-1^) to 10 mL of 50 mM Tris–HCl buffer (pH 8.6). Following a 5 min sonication treatment to disperse the g-STO:Rh particles, measurements were performed at room temperature. Amino acids (tryptophan, serine, tyrosine, threonine, and histidine) were individually dissolved in 50 mM Tris–HCl buffer (pH 8.6) at a final concentration of 1 mM, and measurements were conducted at room temperature.

### Diffuse reflectance absorbance measurement

Diffuse reflectance absorbance measurements were performed using a UV–Vis spectrophotometer (UV-2600i, Shimadzu). Diffuse reflectance spectra of g-STO:Rh and g-STO samples were obtained in the spectral range of 300–800 nm at 25 ºC. g-STO:Rh and g-STO samples were measured using a UV–Vis spectrophotometer without dilution.

## Results and discussion

### Analysis of g-STO:Rh effects on amino acids

**Fig. 1:**
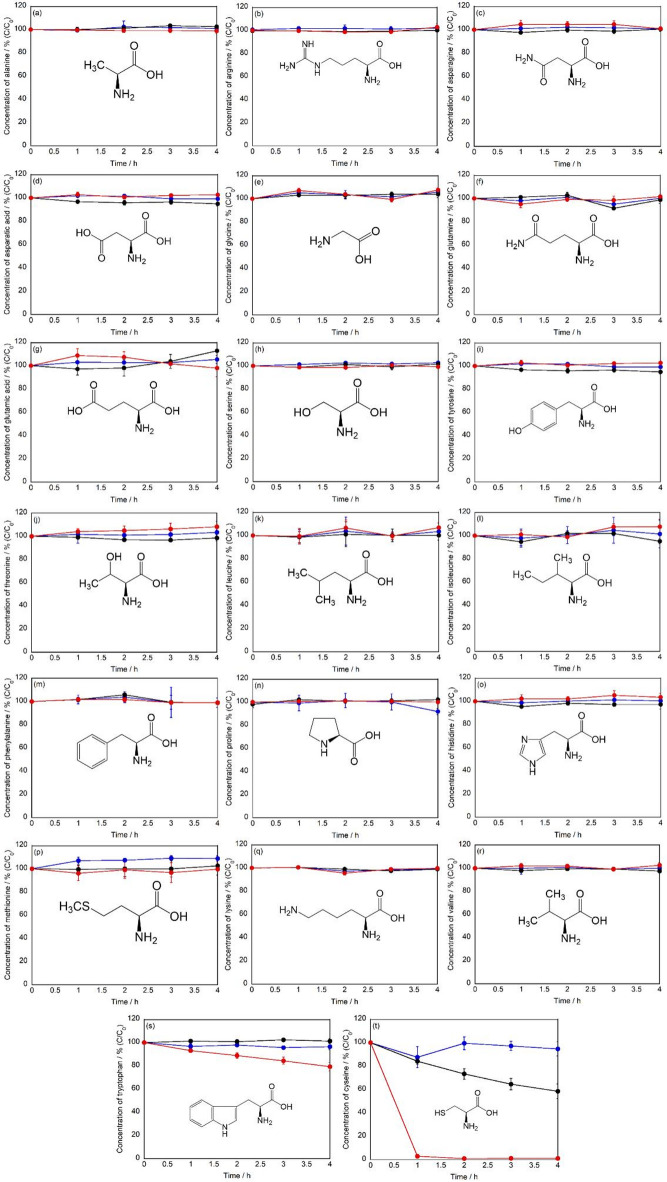
Time course of residual rates of (**a**) l-alanine, (**b**) l-arginine, (**c**) l-asparagine, (**d**) l-aspartic acid, (**e**) glycine, (**f**) l-glutamine, (**g**) l-glutamic acid, (**h**) l-serine, (**i**) l-tyrosine, (**j**) l-threonine, (**k**) l-leucine, (**l**) l-isoleucine, (**m**) l-phenylalanine, (**n**) l-proline, (**o**) l-histidine, (**p**) l-methionine, (**q**) l-lysine, (**r**) l-valine, (**s**) l-tryptophan, and (**t**) l-cysteine (n = 3). Red lines indicate treatment with g-STO:Rh under light irradiation, black lines represent treatment with g-STO:Rh under dark conditions, and blue lines show treatment with light irradiation in the absence of g-STO:Rh.

The photocatalytic effects of g-STO:Rh on all 20 protein-constituent amino acids were quantitatively evaluated using LCMS (Fig. 1). The residual rate is defined as the ratio (C/C₀) of the concentration after treatment (C) to the initial concentration (C₀) of amino acids. After 4 h of treatment with g-STO:Rh under visible light irradiation, no significant decrease in residual rates (p > 0.05) or formation of products was observed for alanine, arginine, asparagine, aspartic acid, glutamine, glutamic acid, glycine, histidine, isoleucine, leucine, lysine, methionine, phenylalanine, proline, serine, threonine, tyrosine, and valine. Control experiments conducted with visible light irradiation alone or g-STO:Rh under dark conditions also showed no significant changes in residual rates for these amino acids.

In contrast, both tryptophan and cysteine exhibited notable decreases in residual rates under visible light irradiation in the presence of g-STO:Rh. For tryptophan, the residual rate significantly decreased to 79.2% after 4 h of light irradiation (p < 0.01). Control experiments showed no significant changes in residual rates, indicating that this decrease was attributable to the photocatalytic effect of g-STO:Rh. For cysteine, a dramatic decrease in residual rate to approximately 1% was observed after 4 h of visible light irradiation in the presence of g-STO:Rh (p < 0.01). Notably, cysteine also showed a significant decrease in residual rate to 58.5% under dark conditions in the presence of g-STO:Rh (p < 0.01). However, the reaction under visible light irradiation demonstrated faster reaction kinetics and a greater decrease in residual rate compared to the dark reaction.

These results demonstrated that the photocatalytic effect of g-STO:Rh exhibits selective reactivity toward tryptophan and cysteine among the 20 amino acids. This selectivity represents a characteristic property that is not observed with conventional photocatalysts such as TiO_2_.

### Analysis of products formed from tryptophan and cysteine following photocatalytic treatment with g-STO:Rh

**Fig. 2:**
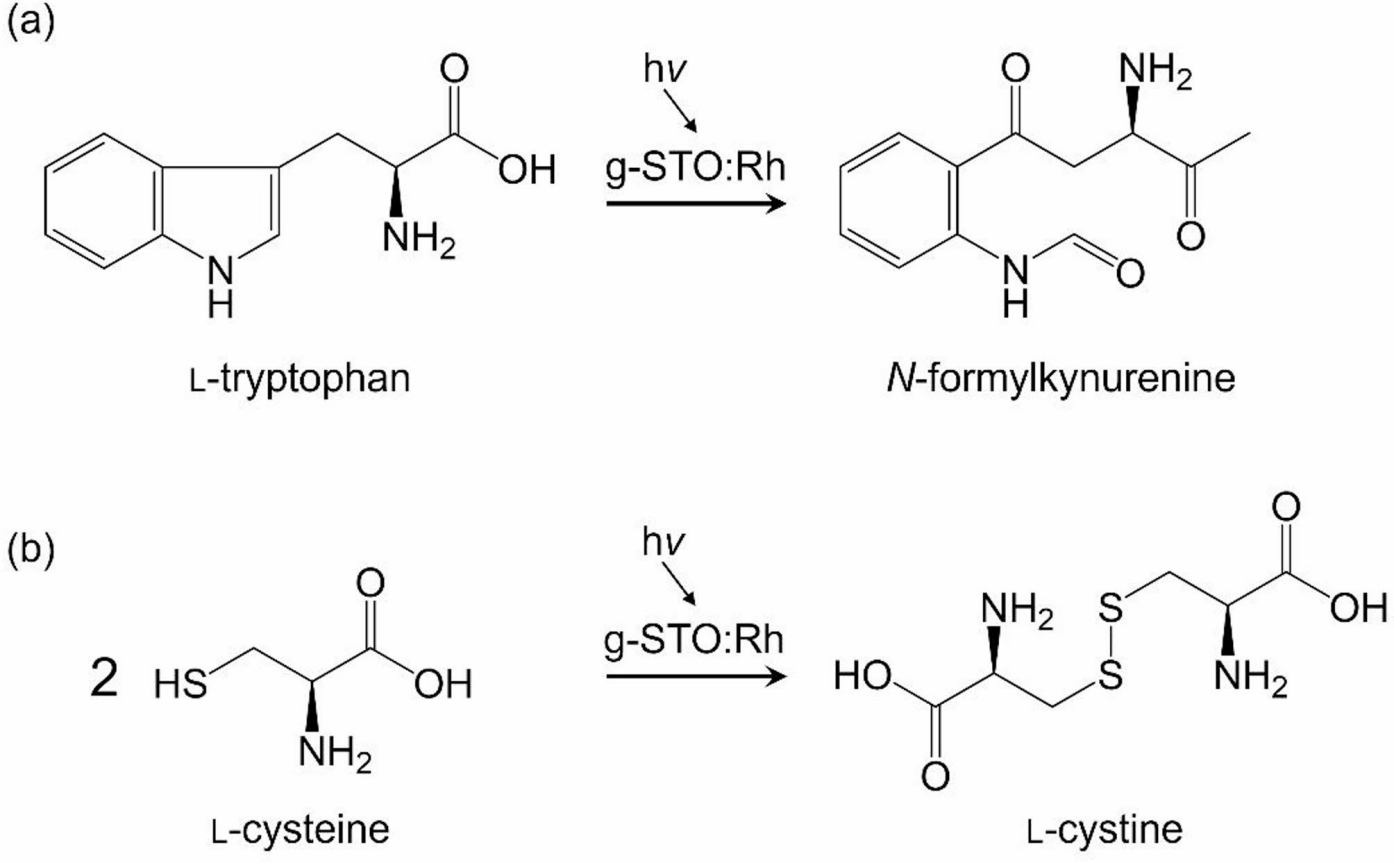
Reaction scheme showing product formation from (**a**) l-tryptophan and (**b**) l-cysteine through g-STO:Rh photocatalytic reaction.

Based on the observed significant decrease in residual rates of tryptophan and cysteine due to g-STO:Rh photocatalytic effects, product identification was performed using LCMS analysis. Mass spectral analysis of the reaction solution after 4 h of light irradiation with g-STO:Rh revealed a new peak at *m/z* = 237 for the tryptophan solution (Fig. S1(a)). This molecular mass corresponded to *N*-formylkynurenine, which was produced through oxidation of the tryptophan side chain (Fig. 2a)^35^. ^1^H and ^13^C NMR spectroscopy was performed to further elucidate the structure of the product with *m/z* = 237. Although signals attributable to impurities originating from the starting material, tryptophan, were detected, characteristic peaks assignable to *N*-formylkynurenine were also observed (Fig. S2). The structure of *N*-formylkynurenine was confirmed and attribution of peaks in NMR was performed using the Yeast Metabolome Database (YMDB)^36^. These results strongly support the identification of the *m/z* = 237 species as *N*-formylkynurenine. Notably, the *N*-formylkynurenine formation from tryptophan was not observed in control experiments conducted with either light irradiation alone or g-STO:Rh under dark conditions (Fig. S3). Previous studies have demonstrated that *N*-formylkynurenine forms when reactive oxygen species generated by TiO_2_ photocatalysts oxidize the tryptophan side chain^31^. In the present study, the selective oxidation of the tryptophan side chain by g-STO:Rh likely proceeds through a similar mechanism (Fig. 2a).

For the cysteine solution treated with g-STO:Rh under light irradiation for 4 h, mass spectral analysis detected a new peak at *m/z* = 241 (Fig. S1(b)). This molecular mass suggested the formation of cystine, produced by oxidative dimerization of the thiol group in the cysteine side chain (Fig. 2b)^37^. The formation of cystine was also observed under dark conditions in the presence of g-STO:Rh. This phenomenon is presumably attributable to the catalytic activity of Rh, as reported in previous studies^38,39^. In this mechanism, the thiol group of cysteine initially adsorbs onto the Rh surface and is converted into a cysteine thiolate. The amino group of the thiolate then coordinates with the Rh surface, facilitating the proximity of adjacent thiol groups. These thiol groups subsequently undergo oxidative coupling to form a disulfide (SS) bond. The resulting cystine is finally released from the Rh surface through desorption. The reaction under visible light irradiation exhibited enhanced reaction kinetics and a greater decrease in residual rate compared to the dark reaction, indicating that both Rh catalytic activity and photocatalytic effects contribute to the conversion of cysteine to cystine. Quantitative analysis of cystine was performed following the photocatalytic treatment of cysteine (1 mM) for 4 h under visible light irradiation in the presence of g-STO:Rh. After 2 h of irradiation, the cystine concentration reached approximately 0.5 mM. Considering that the oxidation of two cysteine molecules yields one cystine molecule, this result indicates that nearly all of the cysteine was converted to cystine within 2 h under the applied conditions. The stability of cystine under these conditions was also evaluated. As shown in Fig. S4, no degradation was observed when cystine was irradiated in the presence of g-STO:Rh under visible light, confirming its stability throughout the reaction period. In addition, ion chromatography was employed to assess whether sulfur-containing byproducts, such as sulfuric acid, were generated during cysteine degradation. The chromatogram obtained after 4 h of treatment is presented in Fig. S5. No detectable formation of sulfuric acid was observed. These results collectively suggest that the photocatalytic oxidation of cysteine by g-STO:Rh proceeds predominantly to cystine, with minimal formation of overoxidized or degraded products.

Degradation experiments of tryptophan and cysteine under visible light irradiation were conducted using undoped SrTiO_3_ (g-STO) and TiO_2_. The results are shown in Fig. S6. No decrease in tryptophan was observed when either g-STO or TiO_2_ was used. In contrast, cysteine degradation was confirmed when using either g-STO or TiO_2_; however, the rate of cysteine reduction was significantly lower compared to when g-STO:Rh was employed. These results demonstrate that under visible light irradiation, g-STO:Rh exhibits higher selective activity compared to g-STO and TiO_2_.

**Fig. 3:**
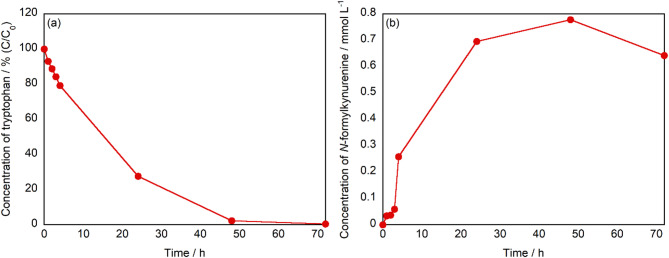
Time-dependent profiles of (**a**) the residual rates of tryptophan and (**b**) the concentration of *N*-formylkynurenine during visible-light irradiation in the presence of g-STO:Rh.

An investigation was carried out to evaluate the oxidation efficiency of tryptophan and the temporal variation in the concentration of *N*-formylkynurenine during extended visible-light irradiation in the presence of g-STO:Rh. The corresponding results are presented in Fig. 3. As shown in Fig. 3a, the residual concentration of tryptophan gradually decreased with increasing irradiation time and was completely decomposed after 72 h. According to Fig. 3b, the concentration of *N*-formylkynurenine reached approximately 0.2 mmol L⁻^1^ after 4 h of irradiation. Given that the decrease in tryptophan concentration at this time point was also around 0.2 mmol L⁻^1^, it is inferred that nearly all of the reacted tryptophan was converted into *N*-formylkynurenine. Following the initial 4-h irradiation period, the concentration of *N*-formylkynurenine continued to increase, peaking at 48 h, and subsequently declined. This decline is attributed to the further decomposition of *N*-formylkynurenine, likely leading to the formation of lower-molecular-weight organic acids such as formic acid and acetic acid, which have been identified in the reaction mixture. The yield and selectivity of *N*-formylkynurenine are summarized in Table S1. The yield increased with irradiation time, reaching a maximum of 64% at 48 h, and then decreased. In contrast, the selectivity was highest at 4 h and gradually declined thereafter.

These results demonstrate that the side chain of tryptophan was selectively oxidized through the photocatalytic effect of g-STO: Rh, while cysteine underwent dimerization.

### Effects of g-STO:Rh on dipeptides

**Fig. 4:**
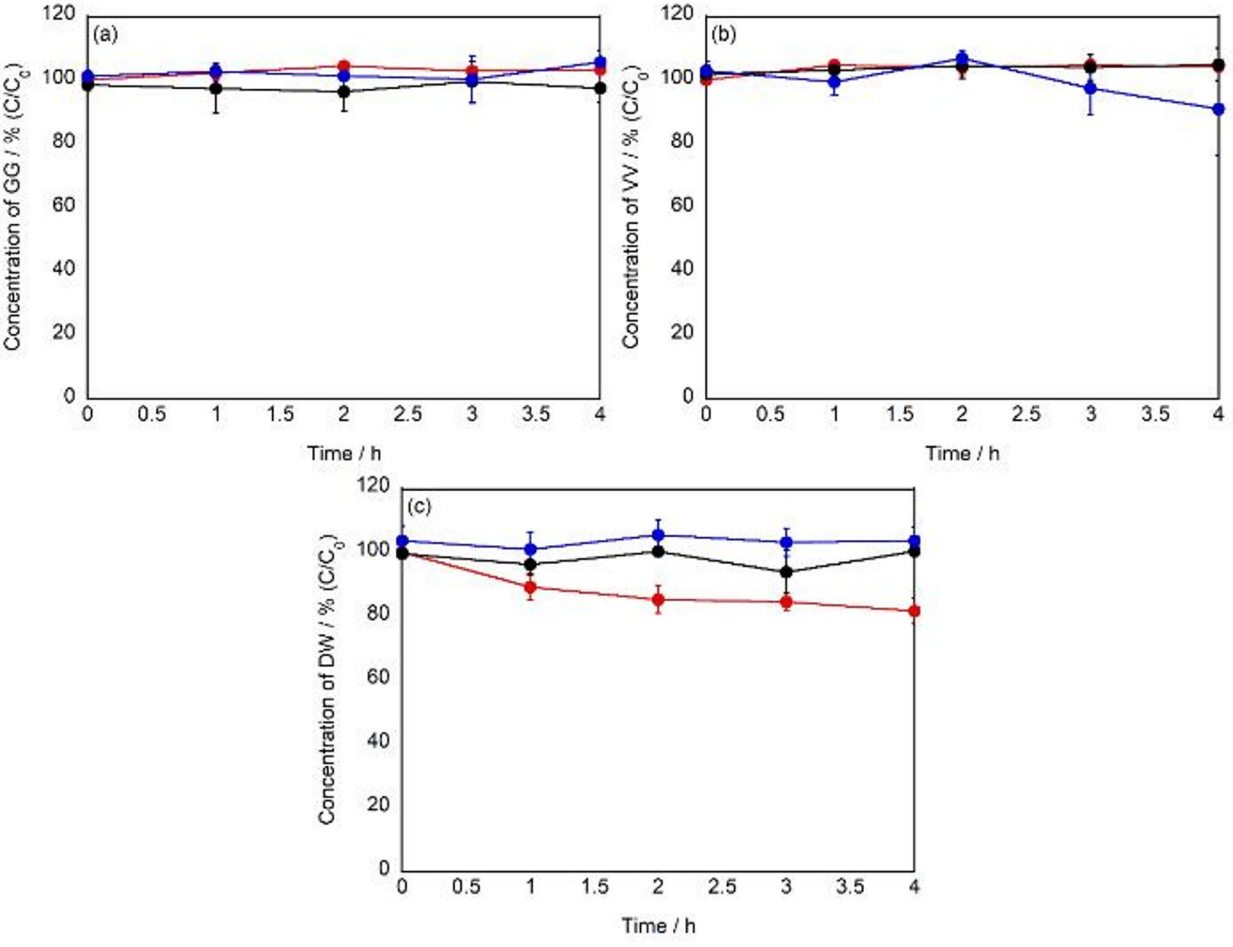
Time course of residual rates for (**a**) GG, (**b**) VV, and (**c**) DW treated with g-STO:Rh under light irradiation (n = 3).

Previous results have revealed that the photocatalytic effect of g-STO:Rh selectively oxidizes the tryptophan side chain to produce *N*-formylkynurenine. To investigate whether this selective side chain oxidation could similarly occur on tryptophan residues in more complex peptide structures, the photocatalytic effects of g-STO:Rh on dipeptides were evaluated. Three dipeptides with different structural features were selected for examination: (i) glycylglycine (GG), a homodimer of glycine without side chain carbons, (ii) valylvaline (VV), a homodimer with branched side chains, and (iii) aspartyl-tryptophan (DW), a heterodimer containing tryptophan and aspartic acid. The experiment using DW aimed to verify whether similar selective oxidation occurs when Trp is bonded to other amino acids. In this study, we consider that adsorption interactions between Trp and the g-STO:Rh surface play an important role in the reaction. Therefore, the type of amino acid adjacent to Trp may change the adsorption efficiency with g-STO:Rh, potentially affecting the oxidation efficiency of the Trp side chain. To test this hypothesis, we deliberately examined whether Trp oxidation occurs under unfavorable conditions. Asp, an acidic amino acid, contains carboxyl groups and exhibits negative charge under physiological conditions. Since the g-STO:Rh surface also possesses a negative charge, electrostatic repulsion makes Asp less likely to adsorb to g-STO:Rh. Therefore, we used peptides (such as DW) where Trp is bonded to the acidic amino acid Asp to verify whether selective oxidation of Trp occurs even in an environment unfavorable for adsorption. When evaluating the residual rates of GG and VV after 4 h of visible light irradiation in the presence of g-STO:Rh, no significant decrease in residual rates was observed for either dipeptide (p > 0.05) (Fig. 4a,b). Similarly, no significant changes in residual rates were observed in control experiments conducted with visible light irradiation alone or g-STO:Rh under dark conditions. These results indicate that the photocatalytic effect of g-STO:Rh does not cause peptide bond cleavage or modification of glycine and valine side chains. In contrast, DW showed a significant decrease in residual rate to approximately 80% after 4 h of visible light irradiation in the presence of g-STO:Rh (p < 0.05) (Fig. 4c). No significant changes in residual rates were observed in control experiments with visible light irradiation alone or g-STO:Rh under dark conditions. This reactivity showed a trend consistent with the previously observed photocatalytic effect of g-STO:Rh on the side chain oxidation of tryptophan alone, suggesting the possibility of selective side chain oxidation occurring on tryptophan residues within dipeptides as well.

### Analysis of products formed from DW treated with g-STO:Rh

**Fig. 5:**
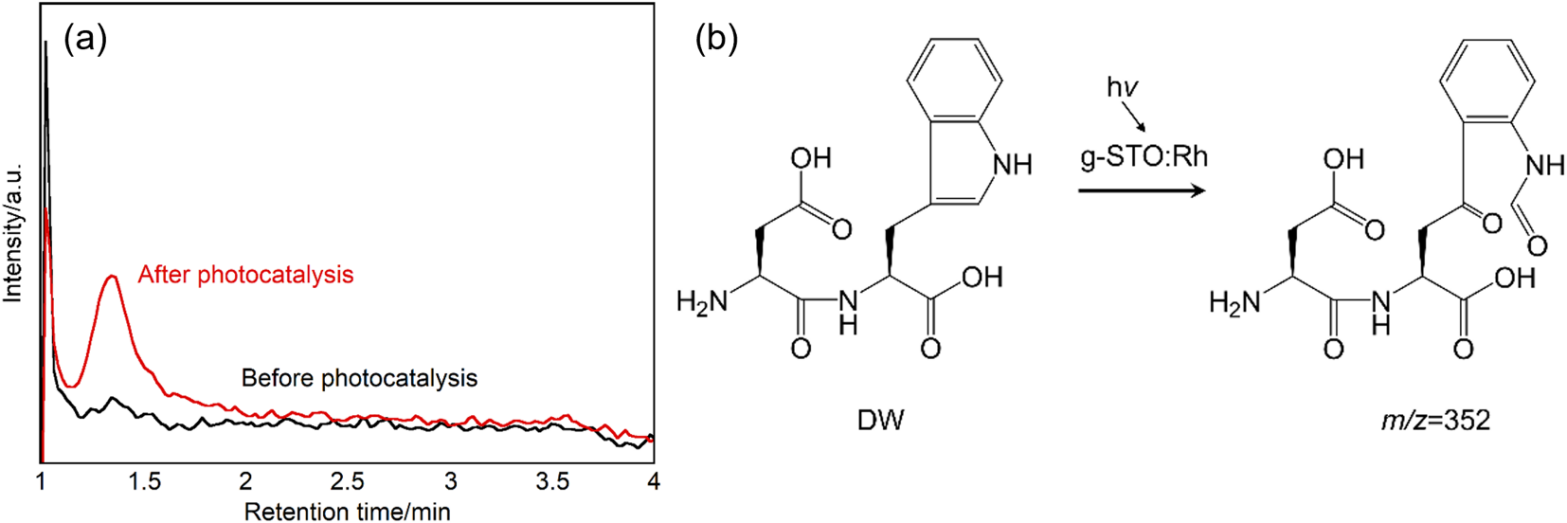
(**a**) Mass chromatogram of *m/z* = 352 obtained from DW treated with g-STO:Rh under light irradiation. Product of *m/z* = 352 corresponded to the proton adduct. (**b**) Reaction scheme showing product formation from DW through g-STO:Rh photocatalytic reaction.

Product identification was performed using LCMS analysis on the reaction solution where DW concentration decreased due to g-STO:Rh photocatalytic effects. Analysis using selected ion monitoring mode detected a new peak at retention time 1.35 min with *m/z* = 352 in the reaction solution after 4 h of light irradiation in the presence of g-STO:Rh (Fig. 5a). This molecular mass corresponds to the theoretical [M + H]⁺ ion value for the product, where the tryptophan residue in DW was converted to an *N*-formylkynurenine residue. This peak was not detected in control experiments conducted with either light irradiation alone or g-STO:Rh under dark conditions. These results demonstrate that the photocatalytic effect of g-STO:Rh selectively oxidized the tryptophan residue within the dipeptide, converting it to an *N*-formylkynurenine residue (Fig. 5b). Combined with the results showing no reaction progression for GG and VV, it became clear that the photocatalytic effect of g-STO:Rh did not affect the peptide bonds themselves but selectively modified the tryptophan residue side chain. This result suggests that g-STO:Rh can specifically recognize and modify tryptophan residues even within peptides.

### Effects of g-STO:Rh on tripeptides

**Fig. 6:**
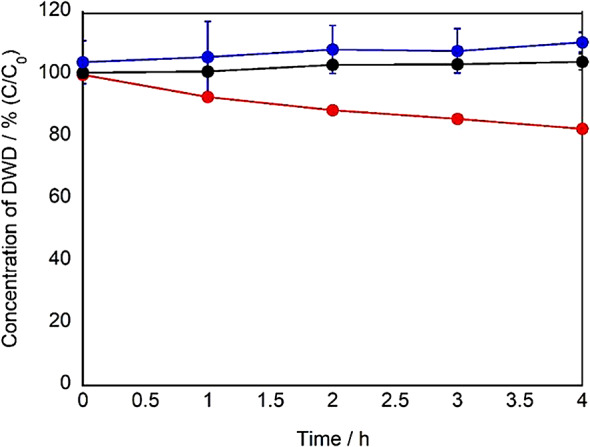
Time course of residual rates for DWD treated with g-STO:Rh under light irradiation (n = 3).

Based on the results from dipeptide studies, the photocatalytic effect of g-STO:Rh was evaluated on longer peptide chains using the tripeptide aspartic acid-tryptophan-aspartic acid (DWD), where tryptophan was positioned in the center. In this experiment, similar to the DW studies, we used peptides (such as DWD) where Trp is positioned between the acidic amino acid Asp to verify whether selective oxidation of Trp occurs even in an environment unfavorable for adsorption. When visible light was irradiated in the presence of g-STO:Rh, the residual rate of DWD significantly decreased to approximately 80% after 4 h (p < 0.01) (Fig. 6). In contrast, control experiments conducted with visible light irradiation alone and g-STO:Rh under dark conditions showed no significant changes in residual rates after 4 h (p > 0.05). These results indicate that the observed decrease in the DWD residual rate can be attributed to the photocatalytic effect of g-STO:Rh. Furthermore, the decrease in DWD residual rate was comparable to that observed earlier with DW (approximately 80% decrease), suggesting that the selective oxidation reaction by g-STO:Rh photocatalytic effect is maintained even in tripeptides where the tryptophan residue is flanked by amino acids on both sides. These results demonstrate that the photocatalytic effect of g-STO:Rh can effectively modify tryptophan residues within longer peptide chains.

### Analysis of products formed from DWD treated with g-STO:Rh

**Fig. 7:**
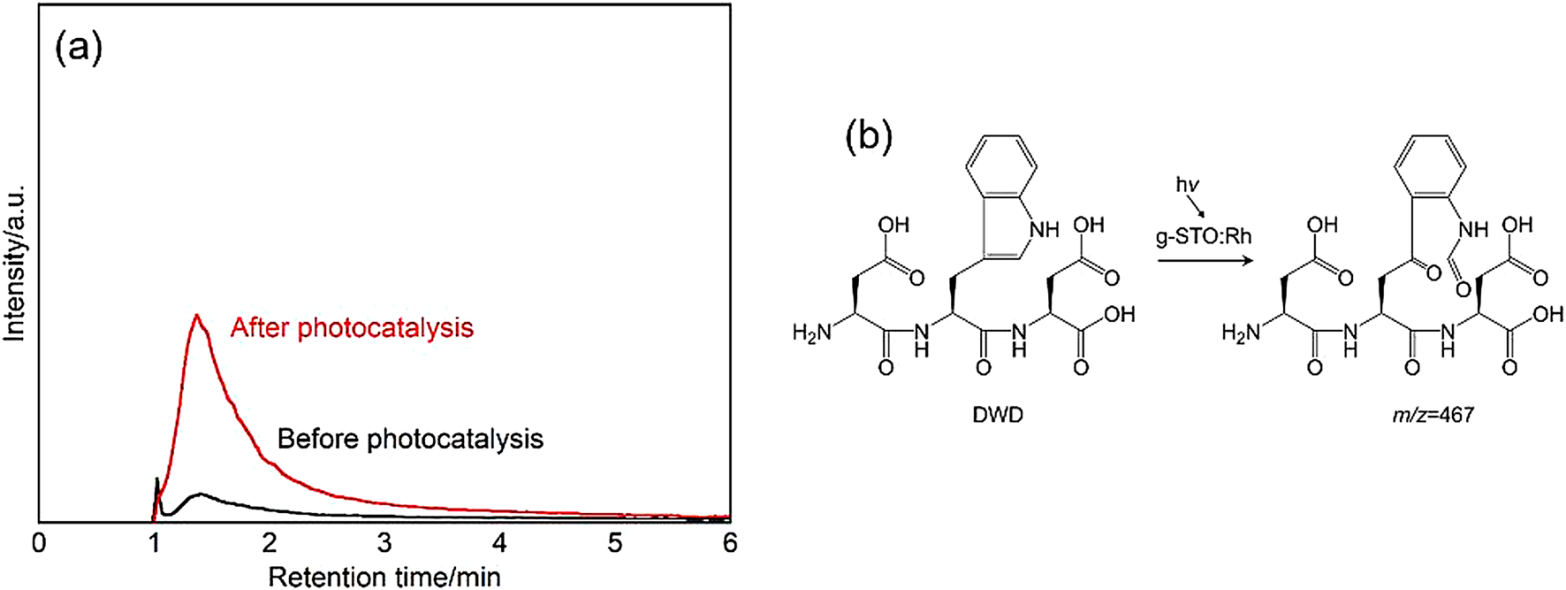
(**a**) Mass chromatogram of *m/z* = 467 obtained from DWD treated with g-STO:Rh under light irradiation. Product of *m/z* = 467 corresponded to the proton adduct. (**b**) Reaction scheme showing product formation from DWD through g-STO:Rh photocatalytic reaction.

Product identification was performed using LCMS analysis on the reaction solution where DWD concentration decreased due to g-STO:Rh photocatalytic effects. Analysis using selected ion monitoring mode detected a new peak at retention time 1.37 min with *m/z* = 467 in the reaction solution after 4 h of light irradiation in the presence of g-STO:Rh (Fig. 7a). This molecular mass corresponded to the theoretical [M + H]⁺ ion value for the product where the tryptophan residue in DWD was converted to an *N*-formylkynurenine residue. This peak was not detected in control experiments conducted with either light irradiation alone or g-STO:Rh under dark conditions. These results clearly demonstrate that the photocatalytic effect of g-STO:Rh selectively oxidized the tryptophan residue within the tripeptide, converting it to an *N*-formylkynurenine residue (Fig. 7b). It is particularly noteworthy that the selective oxidation reaction proceeded efficiently even when the tryptophan residue was positioned between two amino acid residues in the sequence. These findings strongly suggest that selective modification of tryptophan residues by g-STO:Rh is possible even in longer peptide chains.

The selective conversion mechanism of tryptophan was examined from the perspective of amino acid adsorption onto the photocatalyst surface and the subsequent photocatalytic oxidation of amino acids. First, it has been widely reported that adsorption onto the photocatalyst surface plays an important role as the initial step in photocatalytic degradation of amino acids^40–42^. The adsorption process of amino acids onto the photocatalyst surface involves three functional groups: the carboxyl group, amino group, and side chains specific to each amino acid^29^. Although all 20 protein-constituent amino acids possess common carboxyl and amino groups, in this study g-STO:Rh showed significant photocatalytic reactivity only towards tryptophan and cysteine, with no effect on the other 18 amino acids. These results suggested that if interactions with the common structural elements of amino acids (carboxyl and amino groups) were dominant, photocatalytic reactions would have been observed for a broader range of amino acids. Therefore, factors other than these common functional groups are considered to play crucial roles in the photocatalytic activity of g-STO:Rh to amino acids. This inference is consistent with previous findings regarding oxides with terminal OH groups^43–45^. In these oxides, adsorption primarily occurs through –OH and –NH groups in amino acid side chains. For instance, it is known that serine, tyrosine, and threonine with –OH groups in their side chains, or tryptophan and histidine with –NH groups, preferentially interact with terminal OH groups on oxides rather than amino or carboxyl groups^29,46^, showing high degradation rates through photocatalytic effects. In particular, the –OH group in serine’s side chain has been reported to interact strongly with terminal OH of oxides, resulting in high degradation rates^29,47^. Furthermore, infrared spectroscopy measurements have revealed that histidine binds to OH groups on oxide surfaces through the N atom of its imidazole group.

Based on these findings, it was assumed that the photocatalytic effects observed in this study can be attributed to specific interactions between the amino acid side chains and the g-STO:Rh surface. This assumption is also supported by experimental results showing that the decrease in residual rates for DW and DWD was comparable to that of tryptophan alone. This suggests that tryptophan residues in dipeptides and tripeptides are converted through the same mechanism as individual tryptophan. Moreover, regarding the role of photocatalyst surface in this reaction mechanism, Tran et al. reported that terminal OH on solid surfaces plays a major role in effective adsorption of tryptophan, which is consistent with the mechanism proposed in this study^29^.

To better understand these specific interactions, the electrostatic interactions between the photocatalyst surface and amino acids were investigated. Zeta potential measurements of g-STO:Rh showed a negative charge of − 20.5 V. This negative charge is thought to arise from proton dissociation of OH groups on Ti atoms^48^. Zeta potential measurements of various amino acids at pH 8.6 under experimental conditions revealed that tryptophan showed − 7.09 V, indicating a more positive bias compared to other amino acids (serine: − 17.3 V, threonine: − 13.3 V, histidine: − 10.3 V) except for tyrosine (-7.19 V). These results indicate that tryptophan exhibits lower electrostatic repulsion with the negatively charged g-STO:Rh surface compared to other amino acids, suggesting enhanced potential for adsorption.

The oxidation process of amino acids by photocatalysts was further discussed. As mentioned above, tryptophan and tyrosine showed similar zeta potential values, suggesting comparable levels of adsorption onto g-STO:Rh. Since g-STO:Rh generates ·OH radicals, these radicals are probably the primary species involved in amino acid oxidation. In the case of tryptophan, it has been reported that •OH radicals generated by TiO_2_ photocatalytic reactions attack the double bond (between carbon 2 and 3) of the indole ring, leading to pyrrole ring cleavage^31^. Simultaneously, superoxide anions generated during the photocatalytic reaction oxidize these carbon atoms, resulting in the formation of *N*-formylkynurenine^31^. As g-STO:Rh similarly generates both •OH radicals and superoxide anions, it is postulated that the conversion of tryptophan to *N*-formylkynurenine observed proceeds through a similar reaction pathway. Regarding tyrosine, density functional theory (DFT) calculations by Li et al. have shown that •OH radical attack contributes to benzene ring cleavage^49^. In the present study, •OH radicals generated by g-STO:Rh are also likely to attack the benzene ring similarly. However, the photocatalytic oxidation of tyrosine did not proceed effectively, presumably due to the lower reactivity of the benzene ring compared to the indole ring. Fig. S7 presents the diffuse reflectance spectra of SrTiO_3_ (denoted as g-STO) without rhodium doping and g-STO:Rh. The results clearly indicate that g-STO:Rh exhibits distinct absorption bands in the visible light region, in contrast to g-STO. This observation suggests that one of the principal functions of Rh is the extension of the photocatalyst’s absorption range into the visible spectrum. Furthermore, our previous study demonstrated that upon visible light irradiation, the Rh dopants in SrTiO_3_ undergo a transition from the trivalent state (Rh^3^⁺) to the tetravalent state (Rh^4^⁺)^33^. This increase in Rh^4^⁺ is closely associated with the antiphage activity of g-STO:Rh. In a separate study, we reported that g-STO:Rh selectively inactivates the A2 protein of *Qβ* phage^34^, implying that Rh^4^⁺ may play a crucial role in the oxidative degradation of tryptophan, an amino acid component of proteins. Collectively, these findings suggest that Rh contributes not only to the extension of the absorption band into the visible light region but also to the selective oxidation of amino acids through its catalytic redox behavior.

The g-STO:Rh used in this study is an effective photocatalyst for the selective inactivation of Qβ phage. This antiphage activity of g-STO:Rh has been reported to result from selective damage to the A2 protein caused by the photocatalytic effect of g-STO:Rh^34^. From Fig. S4, we observed that excessive degradation of cystine did not occur. Moreover, since cysteine is not contained in the A2 protein^50^, it is considered that neither the oxidation of cysteine nor the oxidative cleavage of disulfide bonds contributes to the antiphage activity of g-STO:Rh. These results suggest that oxidation of tryptophan side chains may be involved as one form of damage to the A2 protein. Therefore, g-STO:Rh potentially possesses the ability to selectively influence protein function.

## Conclusions

In this study, detailed investigations were conducted on the selective modification of amino acids and peptides using the visible light-responsive photocatalyst g-STO:Rh. HPLC and LCMS analyses revealed that the photocatalytic effect of g-STO:Rh exhibited specific reactivity toward tryptophan and cysteine among the 20 protein-constituent amino acids. Through selective oxidation of the indole ring in tryptophan, *N*-formylkynurenine was formed, whereas cysteine underwent dimerization to form cystine. Furthermore, studies on tryptophan-containing dipeptides (DW) and tripeptides (DWD) demonstrated that similar selective oxidation proceeded on tryptophan residues within peptides. The findings from this study indicated that g-STO:Rh specifically recognized tryptophan residues and selectively modified their side chains. This selectivity emerged from specific interactions between the amino acid side chains and OH groups on the photocatalyst surface, which proved crucial in governing the adsorption of amino acids and peptides onto the photocatalyst surface and their subsequent chemical transformation. The methodology established in this study provides a novel approach for selective peptide modification and represents a valuable tool in peptide engineering.

## Electronic supplementary material

Below is the link to the electronic supplementary material.

Supplementary Material 1

## Data Availability

All data included in this study is available upon request by contact with the corresponding author.
