# Supplementary material for: Selective oxidative modification of tryptophan and cysteine residues using visible light responsive Rh doped SrTiO3 photocatalyst
Source: Sci Rep. 2025 Jul 1;15:21697. doi: 10.1038/s41598-025-04870-z (PMC12214781; doi:10.1038/s41598-025-04870-z)
Supplement: Supplementary file 1 — Supplementary Material 1 [file 41598_2025_4870_MOESM1_ESM.docx]

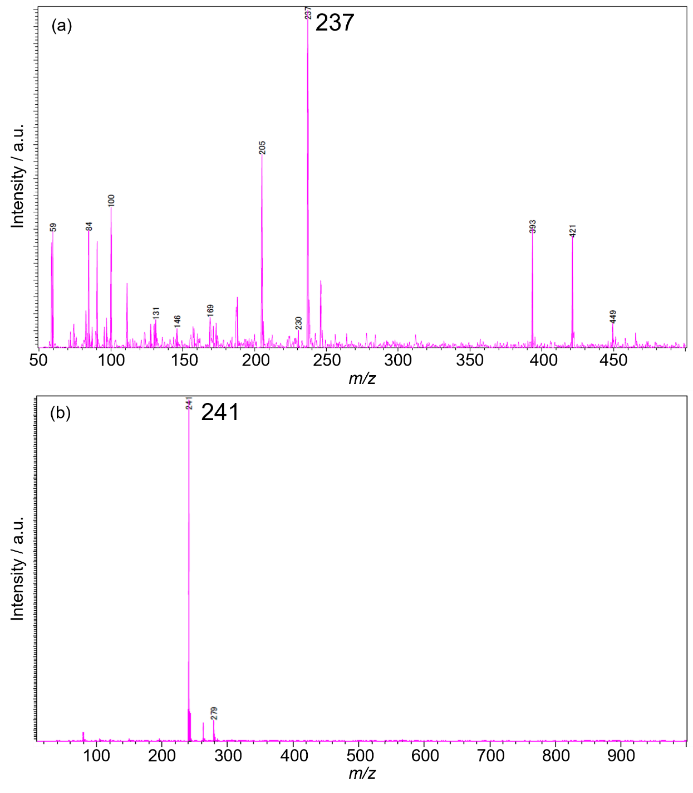


**Fig. S1.** Mass spectra of samples after g-STO:Rh photocatalytic treatment: (a) tryptophan and (b) cysteine


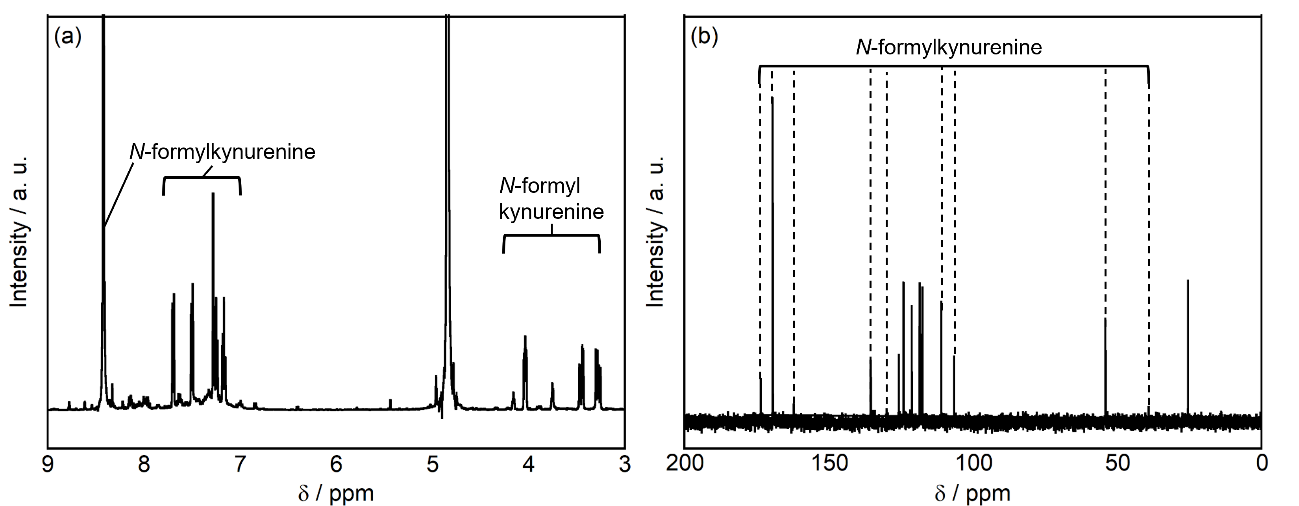


**Fig. S2.** (a) ^1^H and (b) ^13^C NMR spectra of sample after g-STO:Rh photocatalytic treatment. ^1^H NMR δ (500 MHz, D_2_O, 298 K) 8.42 (s, 1H, CHO), 7.70 (d, J=7.5 Hz, 1H, H-6), 7.50 (d, J=8.5Hz, 1H, H-3), 7.30 (m, 1H, H-4), 7.17 (m, 1H, H-5), 4.04 (t, J=5.5 Hz, 1H, H-9), 3.37 (m, 2H, H-8a, H8b). ^13^C NMR δ (125 MHz, D_2_O, 298 K) 39.1 (C-8), 54.1 (C-9), 106. 5 (C-3), 121.2 (C-6), 125.7 (C-1), 130.0 (C-5), 135.4 (C-2), 169.4 (CHO), 173.6 (COOH). The impurity is tryptophan, the starting material.


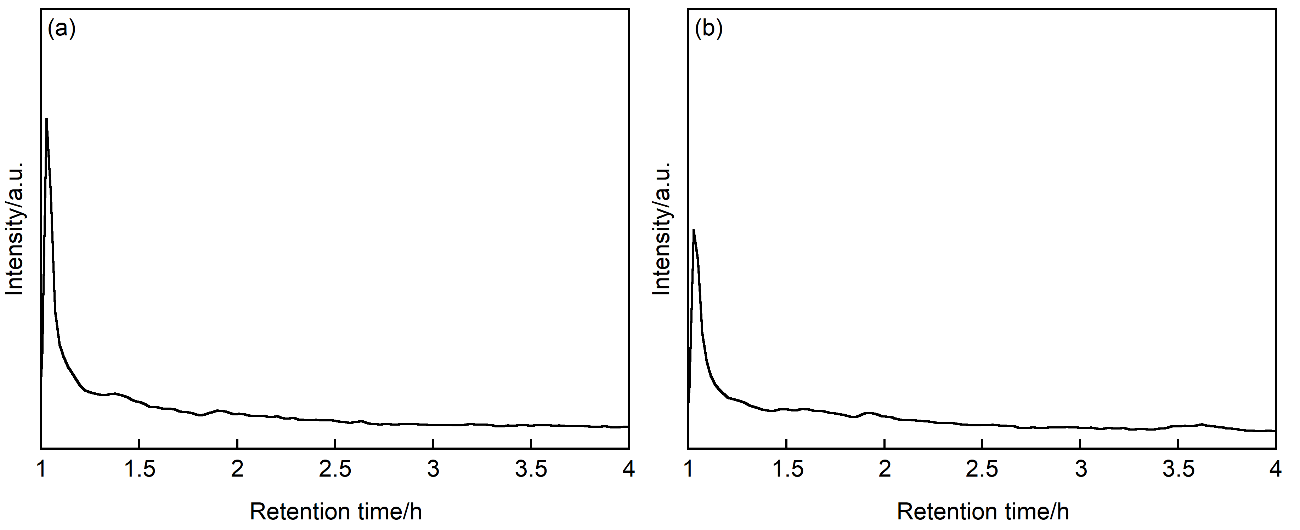


**Fig. S3.** Mass chromatogram of *m/z* = 237 obtained from tryptophan (a) irradiated with visible light and (b) treated with g-STO:Rh in the dark.


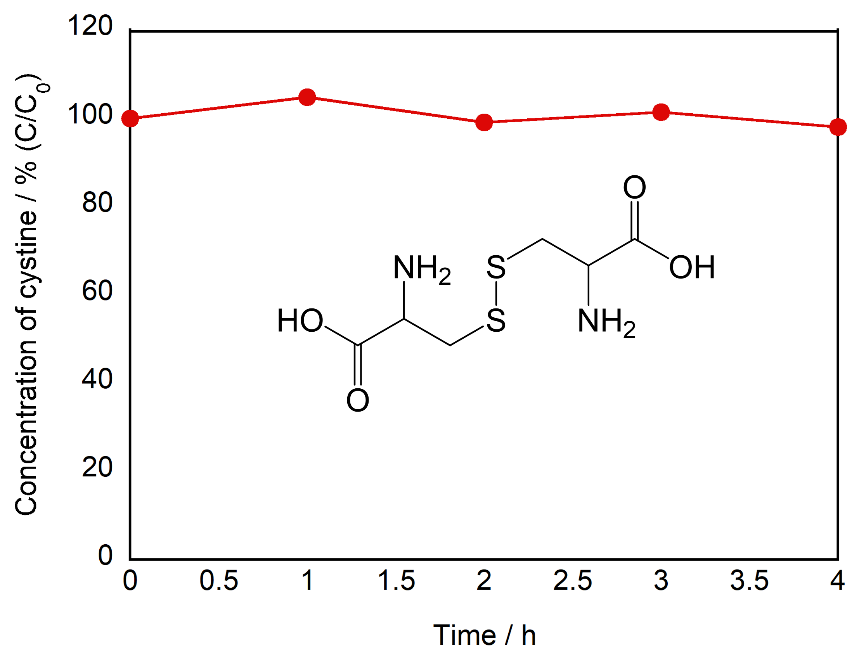


**Fig. S4.** Time-dependent changes in the residual concentration of cystine subjected to visible-light irradiation in the presence of g-STO:Rh.


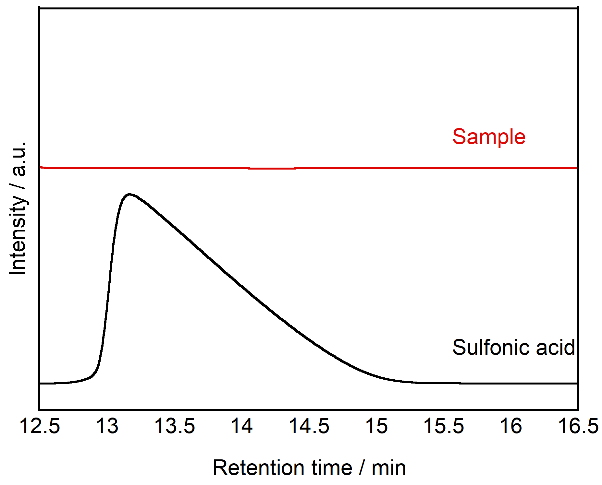


**Fig. S5.** Chromatogram for the sample obtained after 4 hours reaction of cysteine under light (red line) and standard of sulfonic acid (black line).


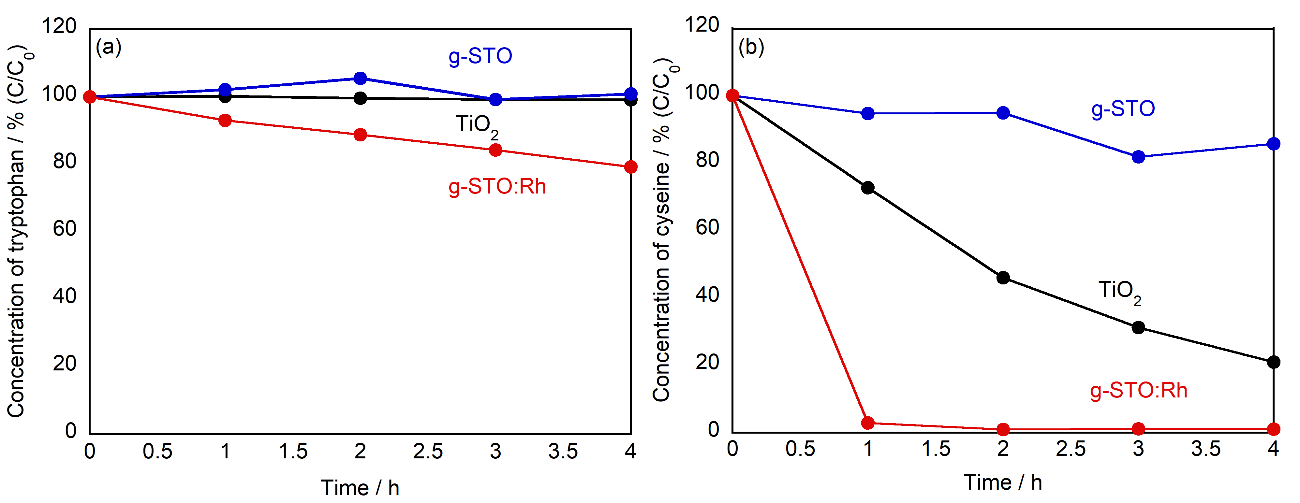


**Fig. S6.** Time-dependent changes in the residual concentrations of (a) tryptophan and (b) cysteine under visible light irradiation in the presence of g-STO (blue) and TiO₂ (black).


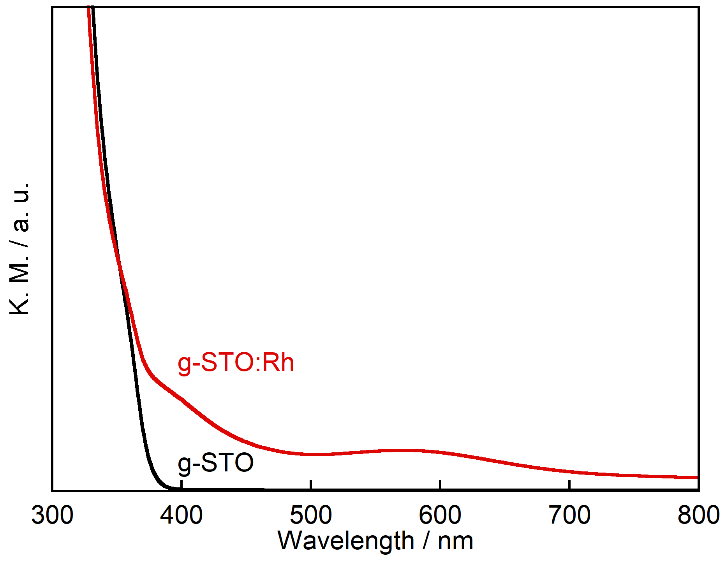


**Fig. S7.** Diffuse reflectance spectra of g-STO:Rh (red) and g-STO (black).

**Table. S1** Yield and selectivity of *N*-formylkynurenine as a function of irradiation time

| Irradiation time / h | Yield / %^†^ | Selectivity / %^††^ |
| --- | --- | --- |
| 0 | 0 | 0 |
| 1 | 3.4 | 48.9 |
| 2 | 3.7 | 32.8 |
| 3 | 5.9 | 37.7 |
| 4 | 25.9 | 99> |
| 24 | 69.5 | 96.2 |
| 48 | 77.8 | 79.7 |
| 72 | 64.2 | 64.5 |

^†^Yield (%) = (concentration of *N*-formylkynurenine)/(initial concentration of tryptophan) × 100

^††^Selectivity (%) = (concentration of *N*-formylkynurenine)/(initial concentration of tryptophan - remaining concentration of tryptophan) × 100
